# Not just passengers: effectors contribute to the assembly of the type VI secretion system as structural building blocks

**DOI:** 10.1128/jb.00455-24

**Published:** 2025-02-04

**Authors:** Sherina Dyrma, Tong-Tong Pei, Xiaoye Liang, Tao Dong

**Affiliations:** 1 Department of Immunology and Microbiology, School of Life Sciences, Southern University of Science and Technology 656360 , Shenzhen, Guangdong, China; University of Notre Dame, Notre Dame, Indiana, USA

**Keywords:** protein secretion, effector, T6SS, contractile sheath

## Abstract

Protein secretion systems are critical macromolecular machines employed by bacteria to interact with diverse environments and hosts during their life cycle. Cytosolically produced protein effectors are translocated across at least one membrane to the outside of the cells or directly into target cells. In most secretion systems, these effectors are mere passengers in unfolded or folded states. However, the type VI secretion system (T6SS) stands out as a powerful contractile device that requires some of its effectors as structural components. This review aims to provide an updated view of the diverse functions of effectors, especially focusing on their roles in T6SS assembly, the implications for T6SS engineering, and the potential of recently developed T6SS models to study effector-T6SS association.

## INTRODUCTION

Once considered a bag of enzymes, bacterial cells do not simply hold their proteins in the bag but have evolved multiple protein secretion systems to release proteins outside for crucial functions. These secretion systems are often required for mediating intra- and inter-cellular interactions in diverse polymicrobial environments, virulence, and survival during pathogen-host interactions (1–3). Therefore, bacterial secretion systems have received tremendous attention and broad interest despite their non-essential role in survival in pure cultures. Among the known systems, most secrete proteins across the double membrane of Gram-negative bacteria through one- or two-step mechanisms (4). One-step systems (T1SS, T3SS, T4SS, T6SS, and T7SS) form trans-membrane tunnels that translocate substrates directly from the cytoplasm to the extracellular environment (5–9). Two-step systems (T2SS and T5SS) use Sec or Tat translocation systems to move effectors across the inner membrane to the periplasm before secretion across the outer membrane (10, 11). These systems secrete diverse effectors involved in stress mitigation, nutrient acquisition, host-microbe interactions, and modulation of polymicrobial communities (12–16).

Effector proteins, as specific substrates of different secretion systems, are recruited through various recognition signal sequences, which are often localized in the C-terminal (T1SS [17, 18]) or N-terminal (T3SS [19] and T4SS [20, 21]) regions of effectors. T6SS-associated effectors lack highly conserved periplasmic translocation (Sec or Tat) signals, but some contain distinct N-terminal signature motifs (22–25). Effector secretion can occur either individually or simultaneously with multiple effectors (11, 26). These multiple effectors may compete for secretion and affect the assembly of the secretion system (27–32). Additionally, effectors may be secreted as unfolded or folded proteins depending on the specific systems (1, 33, 34). This diversity in secretion mechanisms makes bacterial secretion systems both a challenging research topic and a promising field for bioengineering to deliver cargo proteins. While most systems transport effector proteins as passengers, the T6SS has been found to require at least some of its effectors for assembly (30, 31, 35).

This review will discuss the diverse functions of T6SS effectors and recent insights from new T6SS models, with a particular focus on the relatively well-studied T6SS systems in the Pseudomonadota phylum. While the T6SSs in Bacteroidota display unique structural and assembly features, these aspects fall outside the scope of this review and have been addressed in previous studies (36–38).

## T6SS IS A WIDESPREAD DUAL-FUNCTIONAL WEAPON

T6SSs are present in over 25% of Gram-negative bacteria, including human and plant pathogens, commensals, and environmental species (39–41). The intense competition in these environments often drives the rapid evolution of T6SS effectors, conferring diverse functions that enable bacteria to outcompete rivals and adapt to stress conditions (42, 43). Additionally, T6SS influences the spatial and temporal organization of bacterial communities through biofilm formation (44, 45), kin discrimination (45–47), and quorum sensing (48, 49). The T6SSs of some bacteria have also been shown to benefit both host-associated and free-living bacteria by increasing stress resistance, antibiotic resistance, and nutrient acquisition (50–53).

The broad functions of the T6SS are supported by a diverse arsenal of secreted proteins, including three core structural components: the hemolysin-coregulated protein (Hcp), the valine-glycine repeat protein (VgrG), and the proline-alanine-alanine-arginine protein (PAAR) (9, 54). Hcp, VgrG, and PAAR form a spear-like structure that can be equipped with additional cargo effectors, diversifying the types of delivered proteins (32, 42, 55). These secreted proteins exhibit diverse biochemical activities, targeting multiple cellular components in both prokaryotic and eukaryotic cells (43, 55–61).

The T6SS presents a highly promising approach for delivering bioactive proteins into diverse cell types. The feasibility of engineering Hcp, VgrG, and PAAR proteins for delivering cargo domains into target cells has been demonstrated (31, 62–66). However, to fully explore the potential of the T6SS for cargo delivery, a thorough understanding of its structural organization, assembly, and effector loading process is required.

## STRUCTURE AND ASSEMBLY

**Fig 1:**
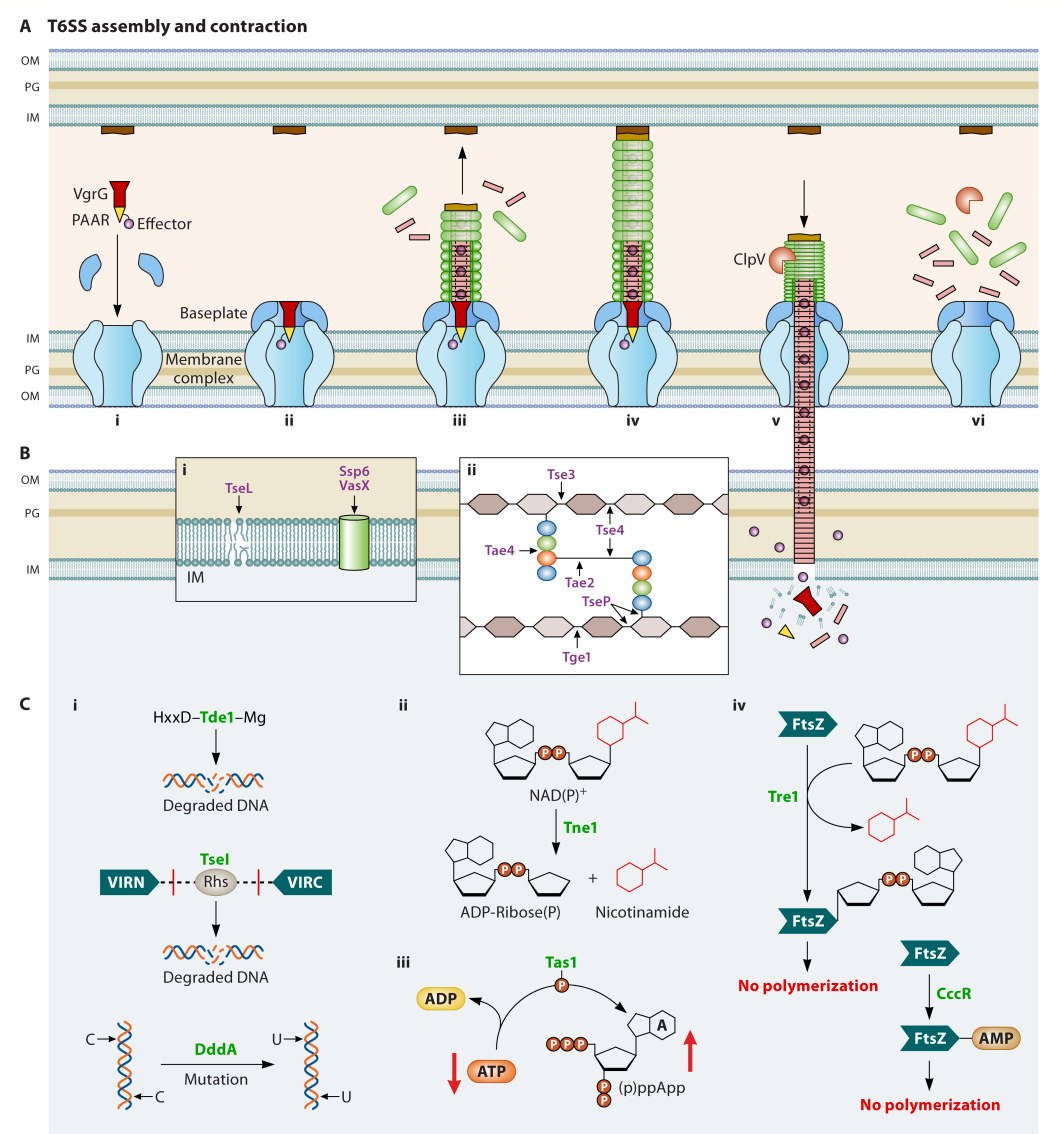
T6SS activity. (**A**) T6SS assembly and construction. (i) The membrane complex forms a transmembrane channel across the periplasm. (ii) The baseplate is recruited to assemble around the VgrG central hub. (iii) VgrG interacts with Hcp to initiate inner tube and sheath assembly. (iv) TssA localized at the distal end of the sheath interacts with TagA that terminates sheath growth. (v) Sheath contraction propels the Hcp inner tail, the VgrG-PAAR central hub, and associated effectors to be expelled outwardly through a drill-like rotational movement. The expelled Hcp-VgrG-PAAR spear breaches the target cell. (vi) ClpV binds to the contracted TssBC sheath subunits to trigger the disassembly of the sheath monomers. The T6SS machinery is disassembled, and monomers are recycled for future assembly. (**B**) Activity of membrane-targeting effectors. (i) Effectors that target phospholipid stability. TseL represents effectors with phospholipase activity that disrupt membrane stability. Ssp6 and VasX belong to pore-forming effectors that create channels in the membrane, leading to loss of membrane potential. (ii) Effectors that target the peptidoglycan. Effectors can target stem peptides for cleavage (Tae4 and Tae2) or the NAG-NAM backbone (Tge1 and Tse3). Dual-activity effectors (TseP and Tse4) can target both stem peptides and the NAG-NAM backbone through endopeptidase and transglycolase activity. (**C**) Activity of cytoplasmic-acting effectors. (i) Effectors that target DNA or RNA. Tde1 and TseI represent two DNAse effectors classes leading to DNA degradation. DddA represents deaminase effectors that cause mutation leading to cell death. TseT represents a class of dual-activity restriction nuclease effectors that cause DNA and RNA degradation. (ii) Tne1 represents NADase effectors that act as NAD(P)+-hydrolases to affect redox balance. (iii) Tas1 is a nucleotidyl-transferase effector that synthesizes ppApp and depletes the ATP pool. (iv) Tre1 and CccR catalyze ADP-ribosylation and AMPylation of FtsZ, respectively, inhibiting cell division.

The T6SS nanomachine of Pseudomonadota species consists of 13 conserved proteins (TssA-M), organized into three primary components: the membrane complex, the baseplate, and the double tubule, which includes an inner tube and an outer sheath (Fig. 1) (9). The membrane complex (TssJML) spans across the periplasm, with the inner membrane protein TssM connecting the outer membrane component TssJ and the inner membrane protein TssL (67–69). The membrane complex acts as a docking site for the baseplate complex. Resembling the baseplates of contractile phages, the T6SS baseplate is structurally a central hub with six wedges and comprises four conserved proteins: TssE, TssF, TssG, TssK, and the VgrG-PAAR spike complex (68, 70). The TssEFGK complex is arranged in a 1:2:1:6 (TssE:TssF:TssG:TssK) stoichiometry organized around the VgrG central spike (71). The VgrG trimeric spike plays a crucial role in initiating the polymerization of the inner tube and recruiting the PAAR protein as its sharp tip (54, 72). While the VgrG spike is essential for T6SS assembly, PAAR is conditionally required depending on the composition of the VgrG spike and its associated effectors (30, 43, 73–76).

The hexameric Hcp tube is surrounded by a contractile sheath made up of TssB and TssC subunits (77, 78). The sheath contracts by expanding radially with a rotational movement, similar to a drill, coupled with vertical compression to propel the inner tube forward (77, 79). Post-contraction, binding sites are exposed to ClpV, a hexameric AAA+ ATPase, which disassembles TssBC from its contracted structure and allows for cytosolic sheath subunit replenishment and reassembly (80).

Some T6SSs also encode ImpA_N domain family proteins, of which TssA is indispensable for T6SS assembly by interacting with multiple structural components and capping the growing distal end of the sheath tube (74, 81, 82). By contrast, another ImpA_N protein is TagA, which acts as a stopper to terminate the sheath-tube growth, and the deletion of *tagA* results in long and curved sheath-tube structures (82–84).

Though not a structural component, the Forkhead-associated (FHA) domain protein also plays a key role in T6SS assembly in multiple T6SSs, some also involving a threonine phosphorylation pathway (TPP) mediated by a kinase PpkA while others do not contain PpkA (85–87). We recently found that Fha primes assembly and forms liquid-liquid phase separation (LLPS) condensates to recruit multiple T6SS structural proteins, Hcp, VgrG, and effector-chaperone pairs (87). These LLPS condensates likely create a locally enriched concentration for more efficient assembly.

## MULTIPLE ROUTES ENSURE EFFECTOR SECRETION

After the T6SS was discovered, one key question was understanding the mechanism of loading effectors. Initially, it was recognized that some VgrG proteins carry extended functional domains that can act as effectors (43), while the Hcp tube can accommodate small effectors inside (88, 89). Later, some PAAR and Hcp proteins were discovered to carry extended effector domains (54, 90–92). These VgrG/PAAR/Hcp proteins with extended domains are also called evolved effectors.

A second class of effectors consists of dedicated effectors without conserved T6SS structural domains, but their secretion is dependent on the cognate VgrG/PAAR/Hcp proteins (32, 55, 61, 89). VgrG-bound effectors were first recognized through genetic screening in *Vibrio cholerae* 7 years after the T6SS discovery (55), and PAAR-bound effectors were found 5 years later (32). With hundreds of effectors discovered to date, the VgrG-PAAR spike-dependent delivery is the most conserved mechanism, whereas the Hcp-tube delivery route is present in a relatively small group of species (32, 43, 88, 89, 93–96).

For some VgrG-PAAR-dependent effectors, chaperone/co-chaperone proteins are required for stability and assembly (32, 93, 95, 97). These chaperone (also known as adaptor) proteins are specific to their cognate toxins and are often encoded adjacent to them, making the conserved chaperone domains valuable tools for identifying novel effectors. While they are not secreted with the effector, a correctly formed effector-chaperone complex is essential for effector loading (32, 97, 98). A few chaperone families have been reported, including DUF1795, DUF2169, and DUF4123 (94, 97, 99). DUF4123-domain proteins are the first reported chaperones mediating loading for VgrG and PAAR-bound effectors (32, 93, 95). A DUF1795-containing chaperone in *Pseudomonas aeruginosa* assists a PAAR-containing effector by covering its transmembrane domains for stabilization (100). A DUF2169-containing chaperone in *Agrobacterium tumefaciens* facilitates the loading of the Tde2 DNase effector (94). The N-terminus of these chaperones likely evolved in response to VgrG and PAAR C-terminal tail differentiation, while their C-terminal ends correlate with the N-terminus of their cognate effectors (32, 94, 95, 97). Some additional chaperone domains, such as DUF 2875 and PRK06147, have also been reported (97, 101, 102).

Although lacking a conserved peptide sequence common for all effectors, several sequence signatures have been reported for T6SS effectors, including MIX (25), FIX (103), and RIX motifs (104), transthyretin-like domains (54), and Rhs (rearrangement hotspot) domains (23, 99, 105). The Rhs core is a cocoon-like barrel enclosing a divergent C-terminal toxin domain (24, 105–107). A release mechanism has been proposed in an *Aeromonas dhakensis* Rhs effector, mediated by autocleavage at the N- and C-terminal ends (24).

## EFFECTOR DIVERSITY

The diversification of T6SS effectors is influenced by evolutionary pressures from environmental stress factors, competition with other microbes, and interactions with predatory eukaryotic cells. Effector acquisition and diversification allow T6SS species to establish complex relationships within their communities, enabling them to target a wide range of organisms including Gram-negative and Gram-positive bacteria, as well as eukaryotes such as fungi, plants, and animals (58–60, 108–111). Discussions on effectors that target eukaryotic cells are available elsewhere (109). When it comes to antibacterial effectors, they can be categorized based on their functional location, either in the periplasm or cytoplasm (Fig. 1; Table S1). A small portion of these effectors has extracellular targets, which facilitate acquiring nutrients or influencing quorum-sensing regulation (53, 112, 113).

Effectors delivered to the periplasm can damage the membrane via lipase activity or pore formation. Lipase effectors (Tle) include five families: Tle1-4 phospholipases hydrolyze glycerol-fatty acid bonds, while Tle5 hydrolyzes the sn3 position (114). Pore-forming toxins disrupt membrane integrity or create pores for ion disruption. These effectors can act as trans-kingdom effectors, affecting both prokaryotic and eukaryotic cells (31, 115–117). Peptidase effectors (Tpe) may target membrane-bound lipoproteins, cleaving their anchors and causing displacement (118, 119). Additionally, a major group of periplasmic effectors degrades peptidoglycans by hydrolyzing the glycan backbone or peptide sidechains, often resulting in abrupt cell lysis (55, 59, 61, 120, 121).

As the T6SSs can directly deliver effectors into the cytosol of recipient cells, cytoplasmic effectors can target crucial cellular targets, most commonly attacking core components like DNA and interfering with NADH, ATP, and purine biosynthesis (Fig. 1). DNase effectors include HNH-family endonucleases (Pfam:PF01844), deaminase toxins, polymorphic nuclease effectors, and restriction endonuclease-like toxins (Tox-REase) (24, 32, 103, 122). Some effectors exhibit peptidases, NAD-glycohydrolases, ADP-ribosyltransferases, and RelA/SpoT-like nucleotidyltransferases activities (98, 123–125). For example, *P. aeruginosa* employs Tas1 to synthesize (p)ppApp, depleting adenosine nucleotides, and RhsP2, an ADP-ribosyltransferase, to modify tRNA and inhibit protein translation (124, 125).

Notably, a group of T6SS effectors can enter recipient cells in a contact-independent manner, especially for nutrient acquisition. *Burkholderia thailandensis* and *Burkholderia pseudomallei* use T6SS effectors for zinc and manganese acquisition (53). *P. aeruginosa* employs effectors like azurin for copper (126) and ModA for molybdate (112), and its H3-T6SS effector TseF interacts with a quorum-sensing molecule *Pseudomonas* quinolone signal and outer-membrane receptors for iron acquisition (113). *Yersinia pseudotuberculosis* secretes Zn^2+^-binding protein YezP for zinc uptake (127, 128). Another effector in *Y. pseudotuberculosis*, CccR, diffuses into recipient cells as a regulator or a toxin, exhibiting dual functions toward kin or competitor cells, respectively (129).

## EFFECTORS SERVE AS CRUCIAL STRUCTURAL COMPONENTS

**Fig 2:**
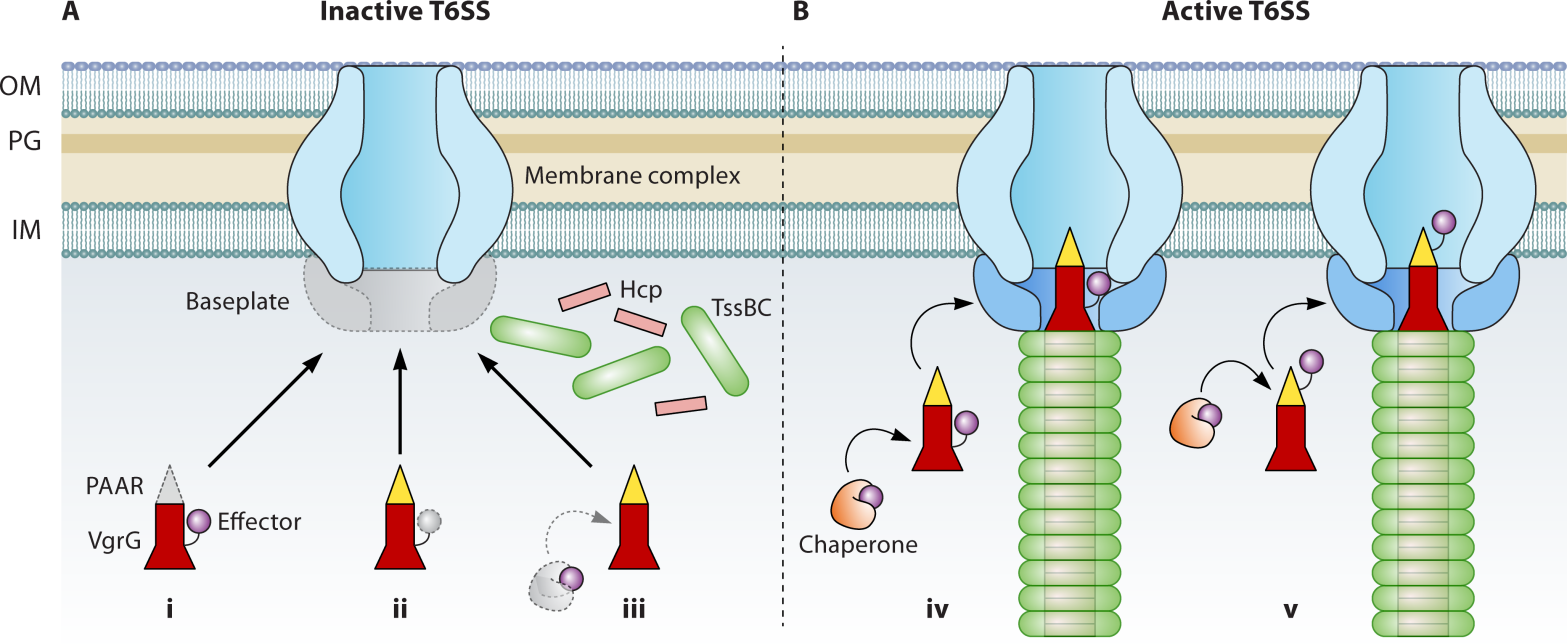
VgrG, PAAR, and associated effectors modulate T6SS assembly. (A) Without PAAR (i), VgrG-associated effectors (ii), and missing chaperones and co-chaperones (iii) may have detrimental effects to T6SS assembly. (B) Assembly of some T6SSs requires at least some effectors to be properly loaded for secretion by associating with the VgrG trimer (iv) or PAAR (v).

As the central hub of the baseplate, VgrG proteins form a trimeric spike essential for T6SS assembly (9, 43, 70). However, most T6SSs encode multiple VgrG paralogs with varying contributions to assembly. Of the three VgrGs in *V. cholerae*, VgrG2 is required, while VgrG1 and VgrG3 are dispensable (43). However, a double knockout of *vgrG1vgrG3* abolishes the T6SS, indicating the need for a heterologous VgrG trimer (130). Similarly, in *A. dhakensis*, although none of the three VgrGs is essential, any double knockout of *vgrG* genes results in a defective T6SS (30). In contrast, in the H1-T6SS of *P. aeruginosa*, VgrG1a is necessary to support assembly, while VgrG1b/1c is dispensible, showing a homologous VgrG1a homotrimer is sufficient (96, 131). What accounts for such variability remains elusive, but recent findings suggest that VgrG-bound effectors and PAAR proteins play a significant role (Fig. 2).

In human pathogens *V. cholerae* and *A. dhakensis* and the plant pathogen *A. tumefaciens*, combinatorial effector gene deletion abolishes T6SS assembly, indicating a conserved requirement for effectors across divergent T6SSs (30, 31, 35). In *A. dhakensis*, swapping the VgrG tail allows a hybrid VgrG homotrimer to secrete two different effectors, bypassing the need for a heterologous VgrG spike (30). This suggests that heterologous effectors dictate the requirement for a heterologous VgrG trimer.

Additionally, chaperones that facilitate effector secretion are also crucial, as combinatorial deletion of chaperone genes severely impairs T6SS secretion, similar to cognate effector deletions (30, 75). These findings suggest that without effectors loaded in place, the T6SS avoids wasting the ejection of hundreds of the carrier Hcp proteins, representing an onboarding sensing mechanism for increased fitness (Fig. 2). A similar cost-saving mechanism is observed when the T6SS structure is defective; genes encoding secreted proteins, but not structural proteins, are down-regulated via an inhibitory regulation triggered by the accumulation of intracellular Hcp, indicating multiple sensing mechanisms for modulating T6SS efficiency (132).

Why are effectors required for assembly? Several clues suggest that effectors may stabilize the T6SS assembly either directly or through the VgrG spike. In a stable non-contractile sheath model in *V. cholerae*, the T6SS structure can assemble without effectors but not in the absence of VgrG proteins (74). In both *V. cholerae* and *A. dhakensis*, effectors directly interact with baseplate components and the key TssA chaperone (30).

Further evidence supporting the role of effectors in stabilizing the T6SS assembly comes from the effects of PAAR proteins (Fig. 2). Although PAAR is a conserved component between the T6SS and the contractile phage, its role is very similar to effectors in the T6SS assembly. PAAR is a monomeric protein sitting atop the flat tip surface of the VgrG trimer, while monomeric effectors attach to the flexible tail on the side of the VgrG trimer (54). PAAR is essential for secretion in some T6SSs (e.g., *Acinetobacter baylyi* and *Serratia marcescens*) but dispensable in others (e.g., *V. cholerae* and *A. dhakensis*) (30, 54, 75, 76, 133). In both *V. cholerae* and *A. dhakensis*, PAAR exhibits VgrG-dependent specificity and essentiality for the T6SS (75). This conditional requirement mimics the effect of VgrG-dependent effectors, supporting a model of VgrG-interacting effectors stabilizing T6SS assembly (30).

## IMPLICATIONS FOR T6SS ENGINEERING

**Fig 3:**
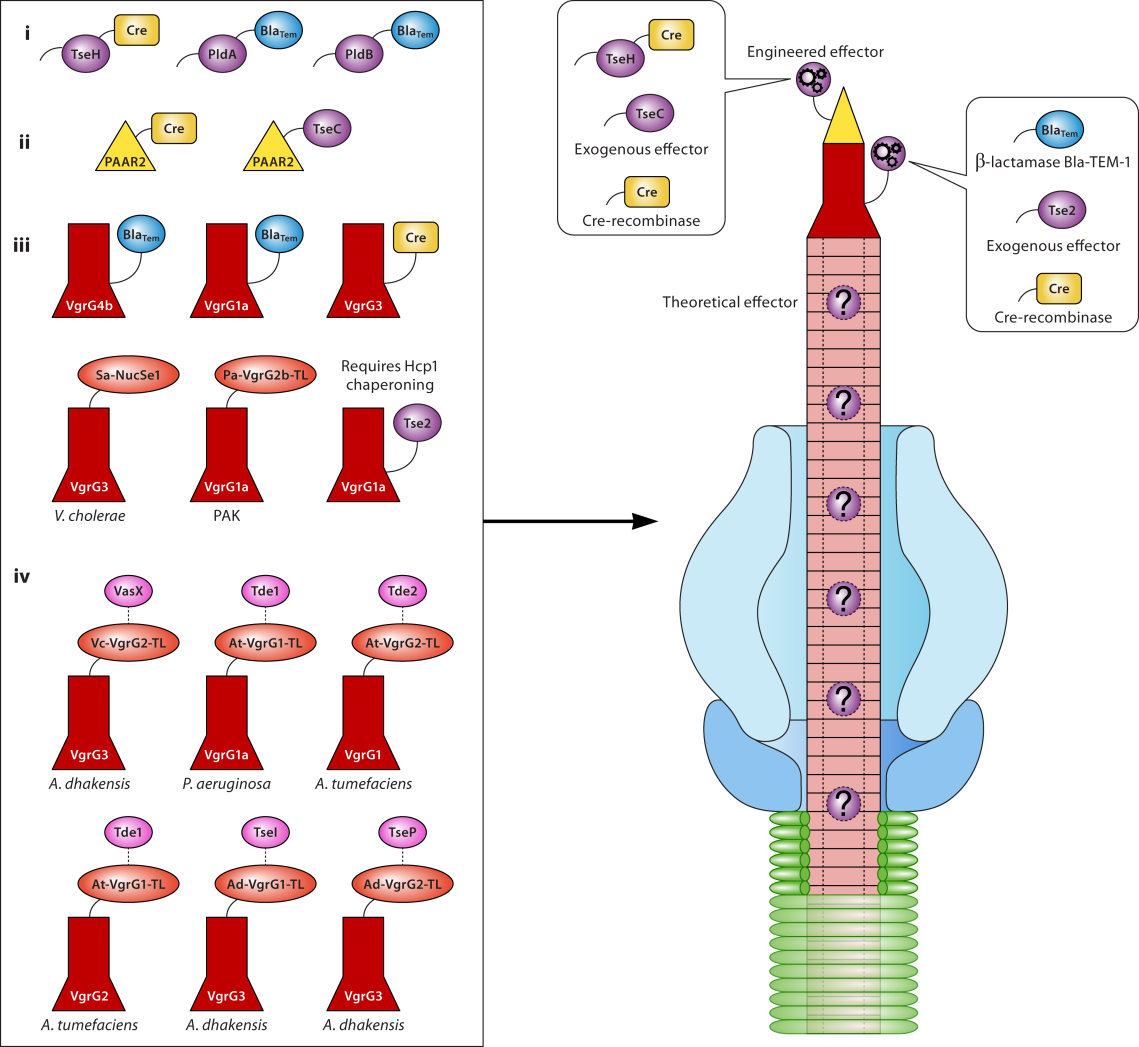
Multiple engineered strategies for cargo delivery. (i) Experimentally validated effectors engineered to deliver cargo domains. (ii) PAAR engineered to deliver novel proteins or heterologous T6SS effectors. (iii) VgrG engineered to deliver novel proteins, heterologous effectors (Sa-NucSe1: protein fusion with *Salmonella* effector NucSe1), or heterologous active VgrG domain through tail-swapping (Pa-VgrG2B-TL: *P. aeruginosa* VgrG2b-tail sequence). (iv) Chimeric VgrG outfitted to deliver heterologous T6SS cargo effectors through tail-swapping (Vc-VgrG2-TL: *V. cholerae* VgrG2-tail sequence; At-VgrG1-TL: *A. tumefaciens* VgrG1-tail sequence; At-VgrG2-TL: *A. tumefaciens* VgrG2-tail sequence; Ad-VgrG2-TL: *A. dhakensis* VgrG2-tail sequence; Ad-VgrG1-TL: *A. dhakensis* VgrG1-TL). Theoretically, Hcp and Hcp-associated effectors can be engineered in a similar manner.

The T6SS has significant potential as a general protein secretion tool due to its ability to deliver multiple effectors into diverse recipient cells. Understanding the structural requirements for effectors is crucial for arming the T6SS with new cargo. Because the T6SS assembly requires the presence of certain effector molecules, the native effectors cannot be entirely swapped out. Instead, they can serve as carriers via fusion with cargo proteins. This strategy has been demonstrated in systems like *V. cholerae* (31, 64, 65) and *P. aeruginosa* (63, 66). Additionally, heterologous cargo delivery can be achieved by swapping the VgrG tail (30, 94) (Fig. 3).

For systems with multiple VgrG-PAAR-effector modules, intracellular competition for association with these structural components should be considered to ensure efficient loading of cargo. In the H2-T6SS of *P. aeruginosa*, the effector TseT requires PAAR4, a chaperone, and a co-chaperone for secretion (32). PAAR4 interacts with multiple VgrG proteins, two of which are needed for TseT secretion. Deletion of another two *vgrG* genes promotes TseT secretion, indicating competition among VgrG-PAAR complexes (32). Competition also exists between PAAR proteins for VgrG binding and between VgrG homologs for baseplate recruitment (30, 75). While deleting competing VgrG homologs may improve secretion, proper stoichiometric amounts of each protein are also required, as overexpression of PAAR or its chaperone TecT interferes with TseT delivery (32). Therefore, comprehensive engineering strategies modulating multiple secretion routes should be considered to ensure efficient cargo delivery.

## NEW T6SS MODELS LEAD TO ADDITIONAL INSIGHTS INTO EFFECTOR ASSEMBLY

Our current understanding about VgrG-dependent effectors and their secretion primarily comes from well-studied T6SS models in *V. cholerae*, the H1-T6SS of *P. aeruginosa*, and *A. dhakensis* (30, 55, 93, 96). Interestingly, all three systems have three VgrG paralogs, providing a simple model for studying VgrG-effector interactions. However, these models fail to capture the complexity of T6SSs with multiple VgrGs and effectors, including the regulation of co-expressed VgrGs, the assembly of heterogeneous VgrG trimers, and the association of effectors in multi-VgrG systems.

Recently, we characterized a powerful T6SS from a plant pathogen *Acidovorax citrulli* AAC00-1 (60, 110). Its genome contains one main T6SS cluster encoding 18 genes (12 core genes and six associated genes) and 13 T6SS-associated clusters encoding 12 VgrG homologs, 17 predicted VgrG-associated effectors, various chaperones, and 1 Hcp. Notably, *A. citrulli* contains 14 Rhs-family effectors (60). Equipped with this large arsenal of effectors, the T6SS of AAC00-1 exhibits strong killing activities against Gram-negative and Gram-positive bacteria, multiple yeast species, and *Mycobacteria* species (60). However, given that each secretion event can accommodate only three VgrG and their associated effectors, how effectors are recruited to the secretion apparatus remains to be elucidated. The features that empower this T6SS with superior killing capabilities against a wide range of prey organisms are also unclear. Nonetheless, it is versatile and complex in the context of VgrG-effectors, while remaining simple due to its constitutive activity and single T6SS system. Therefore, the *A. citrulli* T6SS represents a valuable model for uncovering novel effector functions, exploring VgrG-effector co-evolution, and, perhaps more importantly, understanding how the T6SS coordinates complex cargo delivery networks.

## CONCLUSION AND FUTURE DIRECTIONS

Considering its functional diversity and unique delivery mechanism, the T6SS holds significant potential for development as a general protein delivery tool. To fully unlock this potential, research should be prioritized to two main areas: developing diverse T6SS-active chassis cells for the delivery of select cargo proteins and designing the cargo proteins themselves. Encouraging progress has been achieved in both areas.

Chassis cells refer to engineered cells with specific properties as common platforms for diverse purposes, such as those for cloning and protein purification. Building T6SS chassis cells by genetically modifying the T6SS original host is an effective approach. Detoxifying all T6SS effector activities while preserving delivery capacity has been achieved in *V. cholerae* (31), *A. dhakensis* (30), and the H1-T6SS of *P. aeruginosa* (63), enabling the modified T6SSs to deliver specific cargos effectively. However, to address biosafety concerns and enhance convenience, it would be advantageous to equip commensal strains, such as various *Escherichia coli* strains commonly used for cloning and protein production, with the T6SS. Cloning all T6SS genes on a plasmid has become less challenging, considering the fast development of synthetic biology approaches. However, assembling the T6SS in a heterologous host should not be considered trivial, as it involves a complex task that requires proper expression, folding, and localization of all T6SS proteins to form a functional macromolecular transmembrane apparatus. The heterologous assembly has been successfully demonstrated by expressing the T6SS of *A. dhakensis* in *E. coli* (134) or within the same genus by expressing the T6SS of *Vibrio parahaemolyticus* in *Vibrio natriegens* (135), validating this approach. Alternatively, building T6SS chassis cells by genetically modifying the T6SS original host is also an effective approach. Detoxifying all T6SS effector activities while preserving delivery capacity has been achieved in *V. cholerae* (31), *A. dhakensis* (30), and the H1-T6SS of *P. aeruginosa* (63), enabling the modified T6SSs to deliver specific cargos effectively.

For cargo delivery, we can learn engineering strategies from a common natural feature of T6SS-secreted proteins, including PAAR, VgrG, and effectors: a conserved N-terminus paired with a variable C-terminus (54, 95, 136). For example, the Rhs family features a conserved Rhs core and a divergent C-terminal toxin domain (23, 99). Recently, we identified a dual-functional cell-wall-lysing effector TseP from *A. dhakensis*, comprising an N-terminal amidase and a C-terminal lysozyme. These two domains exhibit significantly different GC (guanine-cytosine) content and can be independently secreted by the T6SS, suggesting an evolutionary fusion event (30, 120). Indeed, building chimeric cargos linked to VgrG, PAAR, and effectors has proven effective, with the successful delivery of beta-lactamase, nuclease effector NucSe1, Cre recombinase, and other heterologous effectors into eukaryotic or bacterial cell cytosol using this strategy (Fig. 3) (31, 62–65, 131). However, it remains challenging to predict the most efficient mode of secretion due to our insufficient knowledge about effector folding and interaction within the baseplate components.

Further research is needed to elucidate the structural organization, recruitment, and sorting mechanisms of T6SS effectors. Advanced imaging techniques like cryo-electron tomography (cryo-ET) to visualize native T6SS structures *in situ* and synthetic biology tools to scale the synthesis of different chimeric modules will likely aid precise model building in the future. Understanding the regulation and interactions of VgrG, PAAR, and associated effectors will enhance our ability to engineer the T6SS for diverse applications, from combating antibiotic resistance to developing novel therapeutic strategies.

## SUPPLEMENTAL MATERIAL

Table S1 jb.00455-24-s0001.pdf T6SS effector functions.

ASM does not own the copyrights to Supplemental Material that may be linked to, or accessed through, an article. The authors have granted ASM a non-exclusive, world-wide license to publish the Supplemental Material files. Please contact the corresponding author directly for reuse.
