# Supplementary material for: Not just passengers: effectors contribute to the assembly of the type VI secretion system as structural building blocks
Source: J Bacteriol. 2025 Feb 4;207(3):e00455-24. doi: 10.1128/jb.00455-24 (PMC11925235; doi:10.1128/jb.00455-24)
Supplement: Table S1 — T6SS effector functions. [file jb.00455-24-s0001.pdf]

Supplemental Table 1 Diversity of experimentally validated T6SS effectors.

	Biochemical Class	Biochemical Activity	Effector	Carrier Protein	Associated Organism	
**Membrane and Periplasmic Effectors**	Glycoside Hydrolase	Hydrolyze MurNAc - GlcNAc glycan strand	Tse3	Hcp1	*Pseudomonas aeruginosa*	1
			Tge1-3		*Pseudomonas protegens*	2
			VgrG3		*Vibrio cholerae*	3
	Amidase	Cleaves peptide bonds as D,L-endopeptidase	Tse1	Hcp1	*Pseudomonas aeruginosa*	4
			Tae1		*Burkholderia thailandensis*	5
			Tae4		*Salmonella enterica*	6
			Ssp1; Ssp2	Hcp1	*Serratia marcescens*	7
		Cleaves peptide bonds as D,D-endopeptidase	Tae2		*Burkholderia thailandensis*	5
			Tae3		*Ralstonia pickettii*	8
	Amidase and Lysozyme	Dual activities: cleaves peptide and glycan bonds	TseP	VgrG2	*Aeromonas dhakensis*	9
			Tse4		*Acinetobacter baumannii*	10
	Metallopeptidase	HExxH zinc-binding motif targets peptidoglycan	VgrG2b		*Pseudomonas aeruginosa*	11
			Tpe1	PAAR	*Acinetobacter baylyi*	12
	Phospholipase	Causes membrane damage	TseL	VgrG3	*Vibrio cholerae*	13
			Tle1-5	VgrG	*Pseudomonas aeruginosa*	14
	Colicin-like	Membrane depolarization leading to pore formation	VasX	VgrG2	*Vibrio cholerae*	15
			TseC	VgrG1	*Aeromonas hydrophilia*	16
			Ssp6	Hcp1	*Serratia marcescens*	17
**Cytoplasmic Effector**	DNase	DNA fragmentation through HxxD motif	Tde1	VgrG1	*Agrobacterium tumefaciens*	18
			Tde2	VgrG2	*Agrobacterium tumefaciens*	18
		NS-2 endonuclease	RhsA	VgrGA	*Dickeya dadantii*	19
		HNH endonuclease	RhsB	VgrGB	*Dickeya dadantii*	19
			TseI	VgrG	*Aeromonas dhakensis*	20
	Polymorphic nuclease	PD-(D/E)xK phosphodiesterase	V12_14465	VgrG1b	*Vibrio parahaemolyticus*	21
	Tox-Rease	Degrades DNA and RNA	TseT	PAAR4	*Pseudomonas aeruginosa*	22
	Deaminase	Causes mutations	DddA	PAAR	*Burkholderia cenocepacia*	23
	NADase	NAD(P)+-hydrolase, redox imbalance	Tne1	PAAR	*Pseudomonas aeruginosa*	24
			Tne2	PAAR	*Pseudomonas protegens*	25
	ADP-ribosyl transferase	Actin ribosylation, cell morphology	RhsP2	VgrG14	*Pseudomonas aeruginosa*	26
			VgrG1		*Aeromonas hydrophilia*	27
			Tre1	PAAR	*Serratia proteamaculans*	28
			Tse2	Hcp1	*Pseudomonas aeruginosa*	29
	Nucleotidyl-transferase	(p)ppApp synthetase, purine synthesis	Tas1	VgrG1	*Pseudomonas aeruginosa*	30
	AMPylation	Filamentation of FtsZ induced by AMP	CccR		*Yersinia pseudotuberculosis*	31
	Metalloprotease	Promotes biofilm formation	TepB	VgrG3	*Pseudomonas aeruginosa*	32
	Ca^{2+} -binding	Ca^{2+} accumulation causes mtROS and mtfragmentation	Hcp2a		*Escherichia coli* (APEC)	33
	DNase and Fe^{2+}-binding	Mg^{2+}-nuclease toxin Proteinaceous Siderophore	TepC		*Yersinia pseudotuberculosis*	34
**Extracellular Effectors**	Proteinaceous siderophores acquire metal ions for stress mitigation	Zn^{2+}-binding	YezP		*Yersinia pseudotuberculosis*	35
			TseZ	Hcp4	*Burkholderia thailandensis*	36
			TseZ		*Burkholderia pseudomallei*	37
		Mn^{2+}-binding	TseM	VgrG4a	*Burkholderia thailandensis*	38
			TseM	VgrG4b	*Burkholderia pseudomallei*	37
			TssS	Hcp4	*Yersinia pseudotuberculosis*	39
		Fe^{2+}-binding	TseF	VgrG3, VgrG1b	*Pseudomonas aeruginosa*	40
			TeoL	VgrG1	*Cupriavidus necator*	41
		Cu^{2+}/Mo^{2+}-binding	Azu	VgrG2b, Hcp2	*Pseudomona aeruginosa*	42
			ModA	VgrG2b	*Pseudomona aeruginosa*	43

References

1. Russell, A. B. *et al.* Type VI secretion delivers bacteriolytic effectors to target cells. *Nature* **475**, 343–347 (2011).
2. Whitney, J. C. *et al.* Identification, structure, and function of a novel type VI secretion peptidoglycan glycoside hydrolase effector-immunity pair. *Journal of Biological Chemistry* **288**, 26616–26624 (2013).
3. Brooks, T. M. *et al*. Lytic activity of the *Vibrio cholerae* type VI secretion toxin VgrG-3 is inhibited by the antitoxin TsaB. *Journal of Biological Chemistry* **288**, 7618–7625 (2013).
4. Chou, S. *et al.* Structure of a peptidoglycan amidase effector targeted to Gram-negative bacteria by the type VI secretion system. *Cell Rep* **1**, 656–664 (2012).
5. Russell, A. B. *et al.* A widespread bacterial type VI secretion effector superfamily identified using a heuristic approach. *Cell Host Microbe* **11**, 538–549 (2012).
6. Zhang, H. *et al.* Structure of the type VI effector-immunity complex (Tae4-Tai4) provides novel insights into the inhibition mechanism of the effector by its immunity protein. *Journal of Biological Chemistry* **288**, 5928–5939 (2013).
7. Srikannathasan, V. *et al.* Structural basis for type VI secreted peptidoglycan DL-endopeptidase function, specificity, and neutralization in *Serratia marcescens*. *Acta Crystallogr D Biol Crystallogr* **69**, 2468–2482 (2013).
8. Dong, C. *et al.* Structural insights into the inhibition of type VI effector Tae3 by its immunity protein Tai3. *Biochemical Journal* **454**, 59–68 (2013).
9. Liang, X. *et al.* Characterization of lysozyme-like effector TseP reveals the dependence of type VI secretion system (T6SS) secretion on effectors in *Aeromonas dhakensis* strain SSU. *Applied and environmental microbiology* **87**, 12 (2021).
10. Le, N.-H. *et al*. Killing of Gram-negative and Gram-positive bacteria by a bifunctional cell wall-targeting T6SS effector. *Proceedings of the National Academy of Sciences* **118**, 40 (2021).
11. Wood, T. E. *et al*. PAAR proteins act as the 'sorting hat' of the type VI secretion system. *Microbiology (N Y)* **165**, 1203–1218 (2019).
12. Ringel, P. D. *et al*. The role of type VI secretion system effectors in target cell lysis and subsequent horizontal gene transfer. *Cell Rep* **21**, 3927–3940 (2017).
13. Dong, T. G. *et al*. Identification of T6SS-dependent effector and immunity proteins by Tn-seq in *Vibrio cholerae*. *Proceedings of the National Academy of Sciences* **110**, 2623–2628 (2013).
14. Russell, A. B. *et al.* Diverse type VI secretion phospholipases are functionally plastic antibacterial effectors. *Nature* **496**, 508–512 (2013).
15. Miyata, S. T. *et al. Vibrio cholerae* requires the type VI secretion system virulence factor VasX to kill *Dictyostelium discoideum*. *Infect Immun* **79**, 2941–2949 (2011).
16. Liang, X. *et al.* Identification of divergent type VI secretion effectors using a conserved chaperone domain. *Proceedings of the National Academy of Sciences of the United States of America* **112**, 9106–9111 (2015).
17. Mariano, G. *et al.* A family of Type VI secretion system effector proteins that form ion-selective pores. *Nat Commun* **10**, 5484 (2019).
18. Ma, L.-S. *et al*. *Agrobacterium tumefaciens* deploys a superfamily of type VI secretion DNase effectors as weapons for interbacterial competition *in planta*. *Cell Host Microbe* **16**, 94–104 (2014).
19. Koskiniemi, S. *et al.* Rhs proteins from diverse bacteria mediate intercellular competition. *Proceedings of the National Academy of Sciences* **110**, 7032–7037 (2013).
20. Pei, T.-T. *et al.* Intramolecular chaperone-mediated secretion of an Rhs effector toxin by a type VI secretion system. *Nat Commun* **11**, 1865 (2020).
21. Jana, B. *et al*. A modular effector with a DNase domain and a marker for T6SS substrates. *Nat Commun* **10**, 3595 (2019).
22. Burkinshaw, B. J. *et al.* A type VI secretion system effector delivery mechanism dependent on PAAR and a chaperone–co-chaperone complex. *Nat Microbiol* **3**, 632–640 (2018).
23. Mok, B. Y. *et al.* A bacterial cytidine deaminase toxin enables CRISPR-free mitochondrial base editing. *Nature* **583**, 631–637 (2020).

24. Whitney, J. C. *et al.* An interbacterial NAD(P)+ glycohydrolase toxin requires elongation factor Tu for delivery to target cells. *Cell* **163**, 607–619 (2015).
25. Tang, J. Y. *et al*. Diverse NADase effector families mediate interbacterial antagonism via the type VI secretion system. *Journal of Biological Chemistry* **293**, 1504–1514 (2018).
26. Bullen, N. P. *et al.* An ADP-ribosyltransferase toxin kills bacterial cells by modifying structured non-coding RNAs. *Mol Cell* **82**, 3484-3498.e11 (2022).
27. Suarez, G. *et al.* A type VI secretion system effector protein, VgrG1, from *Aeromonas hydrophila* that induces host cell toxicity by ADP ribosylation of actin. *J Bacteriol* **192**, 155–168 (2010).
28. Ting, S.-Y. *et al.* Bifunctional immunity proteins protect bacteria against FtsZ-targeting ADP-ribosylating toxins. *Cell* **175**, 1380-1392.e14 (2018).
29. Silverman, J. M. *et al.* Haemolysin coregulated protein is an exported receptor and chaperone of type VI secretion substrates. *Mol Cell* **51**, 584–593 (2013).
30. Ahmad, S. *et al.* An interbacterial toxin inhibits target cell growth by synthesizing (p)ppApp. *Nature* **575**, 674–678 (2019).
31. Wang, D. *et al.* A secreted effector with a dual role as a toxin and as a transcriptional factor. *Nat Commun* **13**, (2022).
32. Yang, Y. *et al.* H3-T6SS of *Pseudomonas aeruginosa* PA14 contributes to environmental adaptation via secretion of a biofilm-promoting effector. *Stress Biology* **2**, 55 (2022).
33. Lu, L. *et al.* Avian pathogenic *Escherichia coli* T6SS effector protein Hcp2a causes mitochondrial dysfunction through interaction with LETM1 protein in DF-1 cells. *Poult Sci* **103**, 103514 (2024).
34. Song, L. *et al.* Trojan horselike T6SS effector TepC mediates both interference competition and exploitative competition. *ISME J* **18**, (2024).
35. Wang, T. *et al.* Type VI secretion system transports Zn^{2+} to combat multiple stresses and host immunity. *PLoS Pathog* **11**, e1005020 (2015).
36. Si, M. *et al.* The type VI secretion system engages a redox-regulated dual-functional heme transporter for zinc acquisition. *Cell Rep* **20**, 949–959 (2017).
37. DeShazer, D. D. A novel contact independent T6SS that maintains redox homeostasis via Zn^{2+} and Mn^{2+} acquisition is conserved in the *Burkholderia pseudomallei* complex. *Microbiol Res* **226**, 48–54 (2019).
38. Si, M. *et al.* Manganese scavenging, and oxidative stress response mediated by type VI secretion system in *Burkholderia thailandensis*. *Proceedings of the National Academy of Sciences* **114**, E2233–E2242 (2017).
39. Zhu, L. *et al.* T6SS translocates a micropeptide to suppress STING-mediated innate immunity by sequestering manganese. *Proceedings of the National Academy of Sciences* **118**, 42(2021).
40. Lin, J. *et al.* A *Pseudomonas* T6SS effector recruits PQS-containing outer membrane vesicles for iron acquisition. *Nat Commun* **8**, 14888 (2017).
41. Li, C. *et al.* T6SS secretes an LPS-binding effector to recruit OMVs for exploitative competition and horizontal gene transfer. *ISME J* **16**, 500–510 (2022).
42. Han, Y. *et al.* A *Pseudomonas aeruginosa* type VI secretion system regulated by CueR facilitates copper acquisition. *PLoS Pathog* **15**, e1008198 (2019).
43. Wang, T. *et al. Pseudomonas aeruginosa* T6SS-mediated molybdate transport contributes to bacterial competition during anaerobiosis. *Cell Rep* **35**, 108957 (2021).
